# Book Review

**Published:** 1893-06

**Authors:** 


*The Extra Pharmacopœia.* By WILLIAM MARTINDALE. Seventh Edition. Pp. viii., 524. London: H. K. LEWIS. 1892.—It is enough to say of a work which is so widely known and has met with such general acceptance by the medical profession that this, its latest edition, is fully up to date in all respects, and that there will be found in it an adequate account of remedies, both new and old.

*Keep your Mouth Shut. A Popular Treatise on Mouth Breathing.* By F. A. A. SMITH, M.D. Pp. 49. London: BAILLIERE, TINDALL, & COX. 1892.—There is a good deal of common sense in this book, and the question of mouth breathing is a very important one. We do not consider, however, that the surgical measures usually necessary for remedying this method of breathing are at all fit for discussion in a "popular treatise;" nor do we think that a book that is professedly written for the laity will be "popular" with the profession.

*A Primer on the Art of Massage (for learners).* By Dr. STRETCH DOWSE. Pp. vi., 151. Bristol: JOHN WRIGHT & Co. 1892.—"A good masseur must understand the primary laws of physiology and of physics." An attempt is made to define the art; the problem to be solved being stated as follows: "The application of sentient living matter to sentient living matter in divers ways, with varying degrees of energy, according to the resistances in the tissues which have to be encountered and overcome." The text is clear, and the illustrations are excellent. If any book can teach the art, surely this one should succeed.

*Epidemic Pneumonia at Scotter.* By T. B. FRANKLIN EMINSON. Pp. 66. London: W. H. AND L. COLLINGRIDGE. 1892.—This history includes four distinct outbreaks dating from April, 1890, when an appalling fatality of 72.2 per cent. was attributed to sewer-air emanations and simple contagion conjointly. The three more recent epidemics bring strong additional evidence in favour of both these points. These epidemics, traced clearly to defective sanitation in rural villages, convey an important lesson to those whom it may concern.

*Alcohol and Public Health.* By J. JAMES RIDGE, M.D. Pp. vi., 91. London: H. K. LEWIS. 1892.—The author is well known to be a powerful advocate of total abstinence: he has long had the conviction that, unless the practice of total abstinence is physiologically right, it cannot be morally binding upon all; but if it be physiologically right, the force of the moral argument must be irresistible. He endeavours to show that the practice is right both on physiological and pathological grounds, and illustrates the effects of alcohol on protoplasm by means of his well-known geranium cuttings, by experiments on the water-flea, and on the hatching of the eggs of the blow-fly. He produces a powerful array of facts in favour of his conclusion.

*The Prescribers' Guide to the Harrogate Mineral Waters.* By H. J. JOHNSTON-LAVIS, M.D. Pp. 48. London: HENRY RENSHAW. 1892.—This small volume presents in a concise form the main points about Harrogate. The author some years since made a tour of English watering places, and discovered that Harrogate possesses a collection of mineral waters not approached by any locality in England, and only surpassed by one in Europe; viz., the volcanic district of Naples. A dozen years' residence amidst the thousands of thermo-mineral springs of Naples and of Southern Italy and Sicily, some thirty journeys across Europe taking in many of its great spas, and trips to Saratoga, the solfataras, geysers, and hot springs of Iceland, give the writer data by which to estimate the value of Harrogate and its waters. It is needless to add that the author has selected for residence the two districts he names as having the finest collections of mineral waters in Europe; viz., Naples in winter; Harrogate in summer. The addresses will be found at the foot of the preface.

*Anæsthetics: Their Uses and Administration.* By DUDLEY WILMOT BUXTON, M.D., B.S. Second edition. Pp. xiv., 222. London: H. K. LEWIS. 1892.—This most excellent practical manual has been much improved and, having been brought up to date in the present issue, will prove a valuable guide to all who wish to learn the best methods of administering anæsthetics. It is written in a cautious and scientific spirit. The author does not dogmatise upon subjects which are *sub judice*, but gives the reader the benefit of his ample experience and observation. We are glad to notice that too much reference is not made to the work of recent commissions. These are all very well as far as they go, but, inasmuch as their experiments are necessarily made chiefly upon the lower animals, their observations are not to be compared in value to those of the clinical expert. The last words have by no means been said yet on the relative safety of the various anæsthetics, but the book before us summarises our present knowledge on the subject with great fairness and accuracy.

*Coca and Cocaine.* By WILLIAM MARTINDALE, F.C.S. Second Edition. Pp. 76. London: H. K. LEWIS. 1892.—This little book treats of the early history and superstitions in regard to coca. The opinions concerning its value held by modern travellers, especially when they have been exposed to cold and excessive fatigue, and also the ill results of its intemperate use, are related in an interesting manner. Its physiological properties and the various ways in which its salts may be used are well summarised. The addition of salicylic acid to the extent of $1\frac{1}{2}$ parts in 1,000 parts of a 10 per cent. solution of hydrochlorate of cocaine is said to be the best way to prevent the growth of fungi in the solution.

*A Manual of the Operations of Surgery.* By JOSEPH BELL, M.D. Seventh Edition. Pp. xvi., 360. Edinburgh: OLIVER AND BOYD. 1892.—The fact that this work has run through six editions is sufficient proof of its appreciation by those for whom it is written, and the new edition will well maintain its reputation. It is written in a plain and easy style, while at the same time it is contained in a small compass, so as to render it suitable for a working text-book in operations on the cadaver. It will not, of course, compare with many other works on operative surgery for fulness of detail, but as a students' text-book or as a ready guide to the house-surgeon it will prove a trustworthy authority.

*A Guide to the Examinations in Sanitary Science.* By HERBERT JONES, D.P.H. Camb. Pp. 99. London: BAILLIERE, TINDALL, AND COX, 1892.—This little book gives intending candidates a *resumé* of the regulations controlling the Examinations for the Diplomas in Public Health or Sanitary Science instituted by the various examining bodies in the United Kingdom. Selected questions serve to show the extent of knowledge traversed by the examinations, while notes on certain of the questions supply useful hints, not always, however, sufficiently complete, towards their solution.

*A Handbook of the Diseases of the Eye, and their Treatment.* By HENRY R. SWANZY, M.B. Fourth Edition. Pp. xv., 536. London: H. K. LEWIS. 1892.—We gladly welcome another edition of this popular textbook, and we recommend it without hesitation to students and practitioners of medicine as one of the best, perhaps the very best, they can use in the study of eye diseases. In it Dr. Swanzy gives—as he has aimed to do—a succinct and practical account of his subject in its most modern aspect; and though he points out the necessity for consulting original papers when complete information is required, we can from experience of the book assert that it often contains information where larger volumes will be found wanting, and that reference will rarely be made to it in vain. Every branch of ophthalmology has been carefully considered, and all that is of importance regarding it finds mention in Dr. Swanzy's book. The illustrations are numerous and graphic; the printing and paper excellent; and the size of the volume convenient for the student's use while engaged in study in the hospital wards or out-patient department.

*Hygiene and Public Health.* By LOUIS C. PARKES, M.D. Third Edition. Pp. xx., 523. London: H. K. LEWIS. 1892.—Dr. Parkes has introduced some new matter into this edition, dealing with smoke prevention, weather observations, influenza and diphtheria, which adds considerably to the value of the book. The manual is now well known as a plainly written and useful introduction to Hygiene, very suitable for students' use, and very trustworthy in its information.

*Golden Rules of Surgical Practice.* By E. HURRY FENWICK, F.R.C.S. Third Edition. Pp. 67. Bristol: JOHN WRIGHT & Co. [1892.]—The author of this little work, who in this edition has disclosed his identity, seems to consider that Surgery is a *very* exact science, for he has drawn up what he calls a "drill-book" consisting of a number of rules beginning with "always" or "never," and strung together in paragraphs, alphabetically arranged under such headings as "Bones," "Chest," "General," etc. We presume, as the book has reached a third edition, that it has met the requirements of some students, but we very much doubt if any useful instruction can be conveyed by a series of short sentences—good and valuable as many of them are—entirely disconnected from one another and unaccompanied by description or explanation.

*From "Œsypum" to "Lanoline."* Pp. 48. London: BURROUGHS, WELLCOME & Co. 1892.—The title of this *brochure* reads very much like a tourist arrangement, say "From Greece to Berlin," or to be more exact, "From Dioscorides to Liebreich." It is interesting, though at the same time somewhat humiliating, to contemplate for how many centuries the human race may have a good thing within its reach, and yet never extract the best out of it. Until quite recently "wool-fat" was prepared in almost the same way as it was among the ancients; it was not until Professor Liebreich made careful investigations into the properties of the fats of animal integument that cholesterin fat was obtained in all its purity. This which is now known as Lanoline is, on account of its penetrative properties, better suited than lard for the basis of ointments. We can speak very favourably of many of the formulæ given, but must take exception to the claim put forward for the utility of the preparation as a vehicle for the administration of remedies in rheumatic and nervous affections.

